# Use of Pralsetinib as Neoadjuvant Therapy for Non-Small Cell Lung Cancer Patient With RET Rearrangement

**DOI:** 10.3389/fonc.2022.848779

**Published:** 2022-02-10

**Authors:** Ning Zhou, Tong Li, Maoli Liang, Fan Ren, Hong Ni, Wei Liu, Tao Shi, Dongbo Xu, Qiusong Chen, Haonan Yu, Zuoqing Song, Lingling Zu, Shuo Li, Song Xu

**Affiliations:** ^1^Department of Lung Cancer Surgery, Tianjin Medical University General Hospital, Tianjin, China; ^2^Tianjin Key Laboratory of Lung Cancer Metastasis and Tumor Microenvironment, Lung Cancer Institute, Tianjin Medical University General Hospital, Tianjin, China; ^3^Department of Respiratory and Critical Care, Tianjin Medical University General Hospital, Tianjin, China; ^4^Department of Respiratory Medicine, Second Affiliated Hospital of Tianjin University of Traditional Chinese Medicine, Tianjin, China; ^5^Precision Medicine Center, Tianjin Medical University General Hospital, Tianjin, China; ^6^Department of Pathology, Tianjin Medical University General Hospital, Tianjin, China; ^7^Department of PET/CT Diagnostic, Tianjin Medical University General Hospital, Tianjin, China

**Keywords:** RET, pralsetinib, locally advanced (stage III) non-small cell lung cancer, neoadjuvant, targeted therapy

## Abstract

RET rearrangements are rare, and occur in 1%-2% of all non-small cell lung cancer (NSCLC) patients. Pralsetinib has a significant anti-tumor effect in patients with advanced NSCLC and a RET rearrangement. Previous studies have confirmed the efficiency of neoadjuvant target therapy for NSCLC. Herein we present a case involving a female patient who was diagnosed with stage IIIA lung adenocarcinoma and harbored a KIF5B-RET rearrangement based on next-generation sequencing. Radiologic downstaging was indicated after pralsetinib treatment. Therefore, a right lower lobectomy and systemic lymphadenectomy were successfully performed. The postoperative pathologic results revealed a response rate of 74% for primary tumor and no residual viable tumor cells were observed in lymph nodes. The tumor, nodes, and metastases (TNM) stage was ypT1cN1M0. The tumor micro-environment (TME) of the primary tumor was also assessed.

## Introduction

The RET gene was identified as a proto-oncogene in 1985 (1). The RET gene is associated with normal embryonic development (2). RET fusions are rare, occurring in 1%-2% of all patients with non-small cell lung cancer (NSCLC) (3). Patients with RET fusions are prone to brain metastases (4). Because RET fusions occur in lung cancer, RET-targeted therapy has been attempted by clinicians. Unfortunately, the efficacy of some multi-targeted tyrosine kinase inhibitors (TKIs) was not satisfactory, including vandetanib, cabozantinib, and lenvatinib (5). Pralsetinib had a significant effect in patients with advanced NSCLC; specifically, the response rate was 61% (6). Pralsetinib was approved by the Food and Drug Administration (FDA) for treating RET fusion-positive NSCLC in 2020. Several studies have verified the availability of neoadjuvant-targeted therapy for NSCLC patients with ROS1, ALK, and epidermal growth factor receptor (EGFR) alterations (7–9); however, there are no reports regarding pralsetinib as neoadjuvant treatment for NSCLC patients with a RET rearrangement.

## Case Report

**Figure 1:**
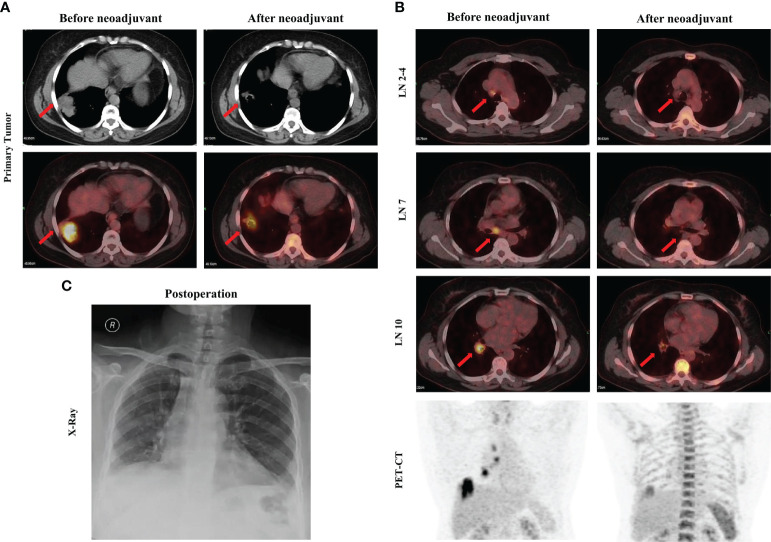
Images before and after neoadjuvant pralsetinib treatment. **(A)** Enhanced CT and PET-CT of the primary tumor. **(B)** PET-CT of the mediastinal lymph nodes and the chest. **(C)** X-ray after surgery.

**Figure 2:**
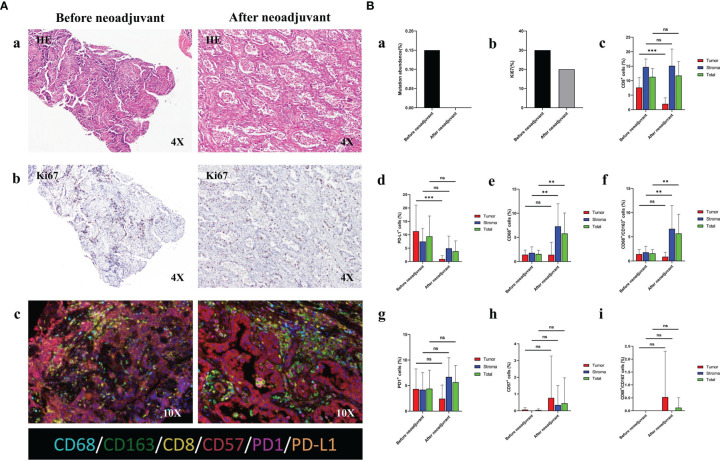
Comprehensive pathological evaluation before and after neoadjuvant pralsetinib treatment. **(A)**. Histochemistry staining before and after neoadjuvant pralsetinib treatment. (a) Hematoxylin and eosin (HE) staining. (b) Ki67 staining. (c) Multiple immunohistochemistry staining on CD68, CD163, CD8, CD57, PD1 and PDL1. **(B)**. Quantitative analysis for plasma ctDNA and staining data. Quantitative analysis for plasma ctDNA (a), Ki67 staining (b), CD8+ (c), PD-L1+ (d), CD68+ (e), CD68+CD163+ (f), PD1+(g), CD57+ (h) and CD68+CD163- (i) cell population. **p < 0.01; ***p < 0.001; ns, not significant.

A 54-year-old female never-smoker was admitted to the hospital for evaluation of a non-productive cough with bloody phlegm for 1 year and persistent chest and back pain for 1 month. Enhanced computed tomography (CT) revealed a mass with a diameter of 42 mm located in the right lower lung with enlarged mediastinal and hilar lymph nodes (stations 2, 4, 7, and 10). The CT findings were confirmed by 18F-fluorodeoxyglucose (FDG) positron emission tomography (PET) as cT2bN2M0, IIIA (AJCC, 8^th^ edition; **Figure 1**). A bronchoscopic biopsy was performed and the pathologic examination revealed that the right lung lesion was a lung adenocarcinoma. We further performed next-generation sequencing (NGS) with a 520-gene panel; a KIF5B-RET fusion was detected. Plasma ctDNA for the RET mutation was also positive with a frequency of 0.15%. After a multiple disciplinary team (MDT) discussion, the patient was diagnosed with a resectable stage IIIA lung adenocarcinoma. Based on the NGS results, we recommended neoadjuvant treatment followed by surgical resection. After obtaining informed consent from the patient, we prescribed pralsetinib at a dosage of 400 mg per day. After 1 month of treatment, a chest CT scan revealed significant shrinkage of the lung tumor (**Figure 1**). A PET-CT scan exhibited significantly decreased F18-FDG uptake in the tumor and no uptake in the hilar and mediastinal lymph nodes (**Figure 1**). In addition, the plasma ctDNA level was also tested, and we showed that the plasma ctDNA was cleared after neoadjuvant treatment (**Figure 2**). During the treatment of pralsetinib, some treatment-related adverse effects were observed, including mild edema and fatigue and moderately increased blood pressure.

Considering that radiologic downstaging was indicated, a right lower lobectomy and systemic lymphadenectomy were successfully performed 1 week after the last dose of pralsetinib (**Figure 1**). Severe adhesions were noted intraoperatively. The postoperative pathologic results showed that although the Ki67 index was significantly decreased, 26% of the tumor cells were still alive in the primary tumor bed however no residual viable tumor cells were observed in lymph nodes. Microscopically, a large number of lymphocytes were infiltrated together with some plasma cells and neutrophils. The lymphatic follicles were formed. Foam cell reactions and cholesterol crystals were observed as well as necrosis and fibrosis. To investigate the change in tumor microenvironment (TME), especially inflammatory and immune cells before and after pralsetinib neoadjuvant treatment, multiple immunohistochemistry (mIHC) staining (Genecast Biotechnology, Wuxi, Jiangsu, China) on the biopsy tissue and surgical sample was performed. The CD68+ CD163+ cell population was significantly increased, whereas the proportion of CD8+ T lymphocytes was decreased (**Figure 2**). PD-L1 expression were also decreased after neoadjuvant treatment (**Figure 2**). The patient received four cycles of pemetrexed (500mg/m^2^, d1) and cisplatin (75mg/m^2^, d1) every 21 days in the adjuvant settings due to the unaffordable economic burden of pralsetinib.

## Discussion

In recent years the benefit of neoadjuvant-targeted therapies for EGFR- and ALK-driven NSCLC patients has been identified (10). To our knowledge, this is the first case of neoadjuvant pralsetinib for NSCLC patients with a RET fusion. In our case pralsetinib exhibited a significant response in a patient with NSCLC and an RET fusion and transformed the unresectable tumor into a resectable tumor; however, 26% of the tumor cells were still alive after pralsetinib neoadjuvant treatment, indicating the necessity of complete resection.

In the current study, we also performed mIHC to determine the changes in TME before and after pralsetinib neoadjuvant treatment. The staining data demonstrated that the proportion of M1 macrophages was upregulated, while the number of CD8+ tumor infiltrating lymphocytes (TILs) and the level of PD-L1 expression were decreased significantly after neoadjuvant treatment. Significant changes in other immune cells, such as natural killer (NK) cells, were not detected. The alteration of these factors indicates that the TME is less inflammatory after pralsetinib neoadjuvant treatment. A previous study suggested that high PD-L1 expression and an increased number of CD8+ TILs are related to clinical benefit in immunotherapy (11). In addition, M1 macrophages are thought to have a direct or indirect anti-tumor role (12). However, in our case, the increased proportion of M1 macrophages and decreased number of CD8+ TILs and PD-L1 expression obscured the role of immunotherapy in subsequent treatment. Further therapeutic strategies after resistance of pralsetinib treatment should be seriously considered.

In conclusion, our case, for the first time suggested that pralsetinib neoadjuvant treatment is feasible for locally advanced NSCLC patients with a RET rearrangement. This patient had an apparent radiologic downstaging after neoadjuvant treatment, which was an indication for complete resection. This case report, however, included one patient only. The role for pralsetinib in neoadjuvant treatment for locally advanced NSCLC and postoperative adjuvant-targeted therapy have not been established for early-stage NSCLC. For NSCLC patients with a RET rearrangement, additional clinical trials are warranted to evaluate the effect of pralsetinib in locally advanced and early-stage NSCLC.

## Data Availability Statement

The original contributions presented in the study are included in the article/supplementary material. Further inquiries can be directed to the corresponding authors.

## Ethics Statement

The studies involving human participants were reviewed and approved by Ethics committee of Tianjin Medical University General Hospital. The patients/participants provided their written informed consent to participate in this study. Written informed consent was obtained from the individual(s) for the publication of any potentially identifiable images or data included in this article.

## Author Contributions

NZ: Data curation, Formal analysis, Writing- Original draft preparation. TL: Formal analysis, Writing- Original draft preparation. ML: Data curation, Writing- Original draft preparation. FR: Formal analysis. HN: Visualization. WL: Resources. TS: Resources. DX: Resources. QC: Resources. HY: Resources. ZS: Investigation. LZ: Investigation. SL: Conceptualization, Validation, Visualization. SX: Conceptualization, Methodology, Supervision, Writing- Reviewing and Editing. All authors contributed to the article and approved the submitted version.

## Funding

The present study was funded by the National Natural Science Foundation of China (82172776) and Tianjin Science and Technology Plan Project (19ZXDBSY00060).

## Conflict of Interest

The authors declare that the research was conducted in the absence of any commercial or financial relationships that could be construed as a potential conflict of interest.

## Publisher’s Note

All claims expressed in this article are solely those of the authors and do not necessarily represent those of their affiliated organizations, or those of the publisher, the editors and the reviewers. Any product that may be evaluated in this article, or claim that may be made by its manufacturer, is not guaranteed or endorsed by the publisher.
